# Predisposing and Precancerous Conditions of the Breast

**Published:** 1904-04-09

**Authors:** 


### Hospital Clinics and Medical Progress.

### PREDISPOSING AND PRECANCEROUS CONDITIONS OF THE BREAST.

In a clinical lecture delivered at St. Bartholomew's Hospital, and reported in the *Clinical Journal*, Mr. Butlin draws attention to the fact that certain so-called simple breast tumours may be the precursors of cancer. Leaving out of consideration the conditions of the nipple and areola which predispose to or which precede cancer, there are, he believes, three affections of the breast in which cancer may supervene, namely:—(1) innocent tumours; (2) duct-cysts; (3) chronic mastitis, with or without the presence of cysts.

With regard to the first of these, Mr. Butlin does not remember to have seen an innocent tumour become malignant, but cases in which such a transformation has undoubtedly occurred have been recorded. In these, the preceding growths have been fibro-adenomata, and the malignant disease has been carcinoma. Sufficient instances of this occurrence are on record to make it undesirable to leave these growths indefinitely on the ground that they are harmless, and he advises their removal within a definite period of time.

In at least two instances Mr. Butlin has seen the development of cancer in connection with duct-cysts. One of these cases was in a lady of 35 years, who came for advice on account of a small cyst of the lower part of the breast. There was no doubt of its nature, for clear fluid could be squeezed out of it through the nipple. The lump had been present for a long time, and appeared to have undergone no change since it was first noticed. No operation was performed. Four and a half years later the patient returned saying that the discharge had become more abundant and was usually blood-stained. On examination the site of the former cyst was occupied by a solid tumour, which proved on removal to be a duct carcinoma. These duct-cysts, then, are certainly a source of danger and should be removed when they are small, when a trivial operation may suffice to rid the patient of a present annoyance and of a future dread.

Probably by far the most common predisposing cause of cancer of the breast is chronic mastitis—with or without the presence of obvious cysts. It must be remembered that there are many lumps formed as a result of chronic mastitis which appear to be quite solid, but which, when they are cut into, are found to contain cysts; and a microscopical examination will often reveal minute cysts which would have quite escaped notice without the help of the microscope. So frequent is the association of cysts with indurations due to chronic mastitis, and so easy is it (with the microscope) to discover the evidence of inflammation in the immediate neighbourhood of single and of multiple cysts, while the intervening tissues are slightly indurated and are the seat of microscopic inflammatory changes, that Mr. Butlin doubts if glandular cysts are not in every instance produced by inflammation. Chronic inflammation of the breast is generally spoken of as if it were confined to the connective tissue only, but it is very rarely that the glandular epithelium is not involved. The tangible signs of chronic mastitis are therefore either indurations or cysts, or a combination of the two. There may be a single cyst, around which there is slight and scarcely perceptible induration. Or there may be more than one perceptible cyst, while the tissues of the breast between the cysts are hardened. Or there may be an ill-defined lump, in which no fluid can be perceived. If it be cut into the lump may be solid, but often it consists of several small cysts and indurated tissue. The diagnosis of chronic mastitis is often impossible. The condition may occur at any period of adult age. It is not in the least limited to the puerperal period, nor does it appear to be so frequent then as later; but it does occur with tolerable frequency at the climateric. Hence the common development of cysts of the breast at that period; hence the term involution cysts, since the latter are regarded as being connected with the involution changes which are in progress at this period, and are therefore looked upon as natural and harmless. The truth probably is that chronic inflammation is most likely to ensue at the time when the breast is undergoing involution, and therefore cysts are most liable to appear at this time. So far from being normal, they are definitely the results of a disease process, and should be regarded as such. Chronic mastitis may affect one breast, and may there form a single tumour or many indurations; or it may attack both breasts, either separately or at the same time. It may also take place in the same breast at different times. Mr. Butlin has seen examples of every one of these conditions. Indurations due to chronic mastitis are much more often multiple than cancers are; they are much more frequently associated with perceptible cysts, they are much more likely to affect both breasts, and they are accompanied by a greater amount of pain in an early stage. Indeed, the attention of the patient is, as a rule, drawn to chronic mastitis by pain in the breast; while cancer is, eight or nine times out of ten, discovered by accident, being quite painless at its commencement. Mr. Butlin doubts if dimpling of the skin ever occurs in connection with chronic mastitis, but the glands in the axilla are frequently enlarged. None of the signs of active inflammation are present, such as increased heat or redness of the skin.

Unfortunately there is no method of distinguishing between the cases which are likely to become cancerous and those which will remain innocent. The treatment which Mr. Butlin advises in young women, and in older women if the condition is of recent occurrence and obviously not malignant, is to firmly support the breast with broad strips of strapping carried round the chest from the spine and ending above and below the other breast. The strapping should be kept applied for a month or six weeks. If a cyst large enough to be diagnosed with certainty be present it should be emptied of its contents by a fine trocar and cannula before the strapping is applied. Tumours which are probably due to chronic mastitis, but which exhibit doubtful signs, especially if they are single and occur in older women, should be incised without delay, so that their real nature may be determined at once. In any case if there is no decided improvement after six weeks of treatment the disease should be removed without hesitation. It suffices to remove the affected portion of the breast, and, where both organs are affected, to remove the affected portion of both, keeping well outside the disease. Mr. Butlin has no sympathy with the advanced doctrine which enjoins the removal of the entire breast in cases of chronic mastitis in which an operation is necessary.

